# Future therapies for cystic fibrosis

**DOI:** 10.1038/s41467-023-36244-2

**Published:** 2023-02-08

**Authors:** Lucy Allen, Lorna Allen, Siobhan B. Carr, Gwyneth Davies, Damian Downey, Marie Egan, Julian T. Forton, Robert Gray, Charles Haworth, Alexander Horsley, Alan R. Smyth, Kevin W. Southern, Jane C. Davies

**Affiliations:** 1 Cystic Fibrosis Trust, London, UK; 2 Royal Brompton & Harefield Hospital, Guy’s & St Thomas’ Trust, London, UK; 3 National Heart & Lung Institute, Imperial College London, London, UK; 4 UCL Great Ormond Street Institute of Child Health, University College London, London, UK; 5 Great Ormond Street Hospital for Children, London, UK; 6 Wellcome-Wolfson Institute for Experimental Medicine, Queen’s University Belfast, Belfast, UK; 7 Yale University, New Haven, CT USA; 8 Noah’s Ark Children’s Hospital for Wales, Cardiff, UK; 9 School of Medicine, Cardiff University, Cardiff, UK; 10 Centre for Inflammation Research, University of Edinburgh, Edinburgh, UK; 11 Western General Hospital, Edinburgh, UK; 12 Royal Papworth Hospital and Department of Medicine, Cambridge, UK; 13 University of Cambridge, Cambridge, UK; 14 Division of Infection, Immunity and Respiratory Medicine, University of Manchester, Manchester, UK; 15 Manchester Adult CF Centre, Manchester University NHS Foundation Trust, Manchester, UK; 16 School of Medicine, University of Nottingham, Nottingham, UK; 17 NIHR Nottingham Biomedical Research Centre, Nottingham, UK; 18 Department of Women’s and Children’s Health, University of Liverpool, Liverpool, UK; 19 Institute in the Park, Alder Hey Children’s Hospital, Liverpool, UK

**Keywords:** Translational research, Cystic fibrosis, Clinical trials, Drug development

## Abstract

We are currently witnessing transformative change for people with cystic fibrosis with the introduction of small molecule, mutation-specific drugs capable of restoring function of the defective protein, cystic fibrosis transmembrane conductance regulator (CFTR). However, despite being a single gene disorder, there are multiple cystic fibrosis-causing genetic variants; mutation-specific drugs are not suitable for all genetic variants and also do not correct all the multisystem clinical manifestations of the disease. For many, there will remain a need for improved treatments. Those patients with gene variants responsive to CFTR modulators may have found these therapies to be transformational; research is now focusing on safely reducing the burden of symptom-directed treatment. However, modulators are not available in all parts of the globe, an issue which is further widening existing health inequalities. For patients who are not suitable for- or do not have access to- modulator drugs, alternative approaches are progressing through the trials pipeline. There will be challenges encountered in design and implementation of these trials, for which the established global CF infrastructure is a major advantage. Here, the Cystic Fibrosis National Research Strategy Group of the UK NIHR Respiratory Translational Research Collaboration looks to the future of cystic fibrosis therapies and consider priorities for future research and development.

## Abstract (web summary)

The cystic fibrosis landscape has changed dramatically over the last few decades, with improvements in patient quality of life, prognosis and predicted survival. In part, this is related to the availability of novel CFTR modulator drugs, although prior advances in symptom-directed therapies and diagnosis had already led to substantial improvements. However, the authors, part of a national CF focused group, recognize that more needs to be done and outline their considerations on research priorities in this perspective.

## Introduction

Cystic fibrosis (CF) affects ~100,000 people globally, with >10,000 on the UK’s patient registry (https://www.cysticfibrosis.org.uk/the-work-we-do/uk-cf-registry/reporting-and-resources). For decades regarded as a fatal inherited disease of childhood, prognosis has improved greatly so that there are now more adults than children in many regions. CF is however still associated with reduced life expectancy and a high treatment burden related to its multisystem manifestations. Many people living with CF now have access to CFTR modulator therapies which, in many cases, have dramatically improved outcomes. Access to these high-cost drugs is not universal, and even in regions where drugs are reimbursed, a proportion of people cannot benefit due to possessing non-responsive gene variants. Here, the CF National Research Strategy group, part of the National Institutes of Health and Care Research (NIHR) Respiratory Translational Research Collaborative review the status of CF today and outline challenges and priority areas for future research.

### The cystic fibrosis landscape today

#### The last few decades: steadily improving outcomes

**Fig. 1:**
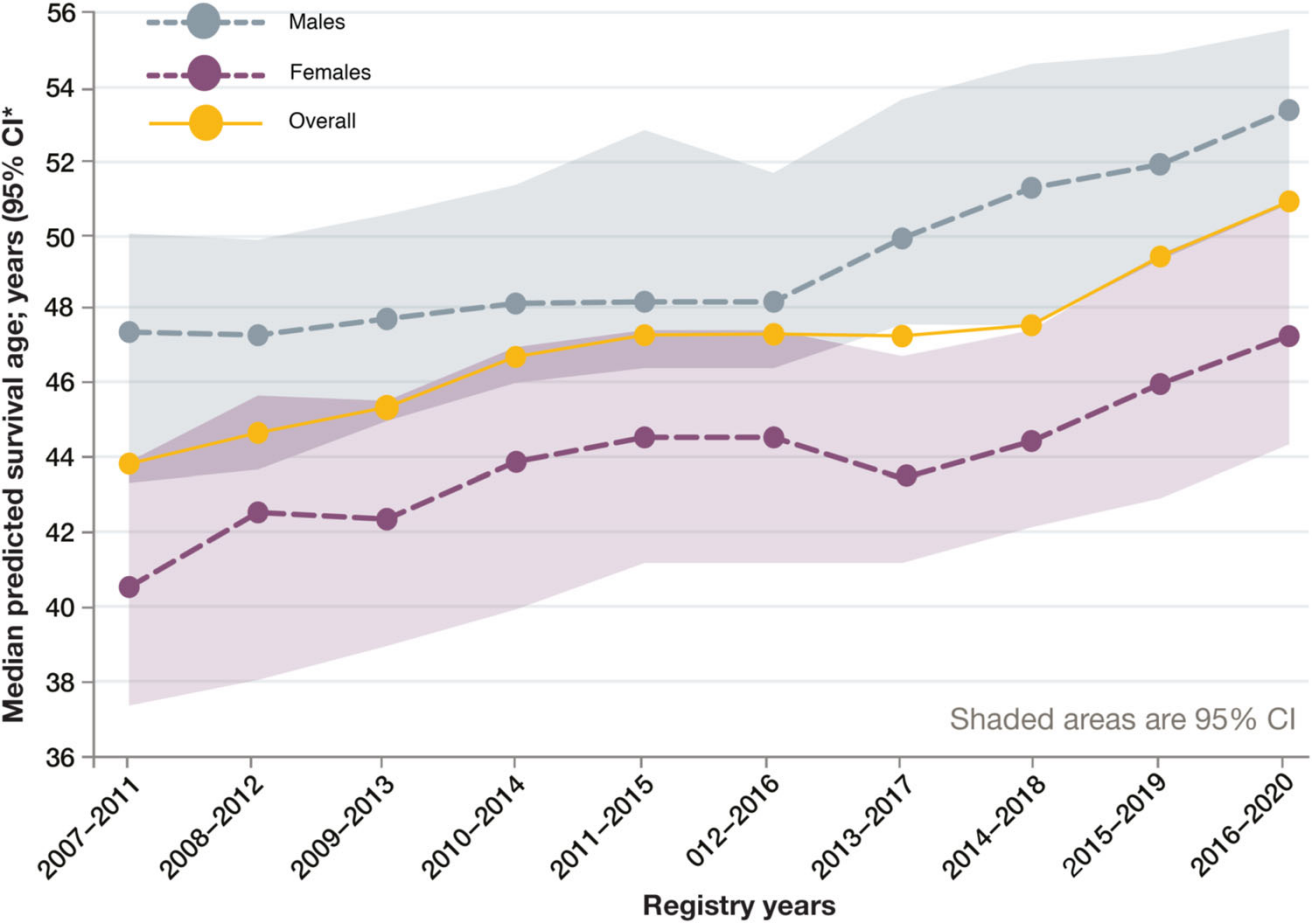
Improvements in median predicted survival in UK pwCF over recent years. From the CF Trust Patient Registry Report (https://www.cysticfibrosis.org.uk/sites/default/files/2022-03/2020%20Annual%20data%20report%20-%20Version%203.pdf).

Many factors over the last few decades have contributed to greatly improved outcomes experienced by people with (pw)CF today (Fig. 1) including (i) timely (early postnatal life) diagnosis, (ii) centre-based, multidisciplinary care delivery models underpinned by a (iii) co-ordinated infrastructure based on (inter)national patient registries, clinical trials networks and partnering patient organisations.

Newborn screening (NBS) programmes, in which heel-prick blood tests are obtained in the first few days of life, have been in place for decades for conditions such as hypothyroidism and phenylketonuria. In the UK and many other countries, these programmes now include CF, leading to a significantly reduced time to diagnosis^1^. Currently, most programmes incorporate DNA analysis as a second stage test to improve the specificity and performance of the protocol and a positive screen will then be confirmed as a CF diagnosis with the gold-standard test, sweat chloride^2^. For the majority of CF infants recognised through NBS, this has been a positive intervention facilitating early access to therapies. There is good evidence that earlier diagnosis has a profound impact on clinical outcomes such as weight gain and survival and is associated with reductions in treatment burden and costs^1,3^.

Long before the recent advances in drug development discussed later in this article, the development of internationally agreed, evidence-based Standards of Care (SoC), underpinned by clinical trials and systematic reviews, drove substantial improvements in clinical outcomes for people with pwCF^4,5^. One major advance was the adoption of centre-based, highly specialised multidisciplinary teams. Respiratory disease remains the predominant manifestation of CF with a progressive decrease in lung function, most commonly assessed with the spirometric measurement of forced expiratory volume in the 1^st^ second (FEV_1_) standardised for height and sex and expressed as percent predicted (pp) of normal. Conventional management strategies^6–10^ include early initiation of airway clearance with physiotherapy and mucus-clearing, inhaled drugs; antimicrobials through a variety of routes; escalation of treatment for episodes of increased chest symptoms (pulmonary exacerbations, PEx); supplementing pancreatic enzymes and fat-soluble vitamins, which are deficient due to pancreatic ductal obstruction, thereby optimising growth and nutrition; and routine surveillance for developing complications such as liver disease, CF-related diabetes and bone disease. The major cause of premature death in CF is lung disease progression with the eventual development of respiratory failure. At this stage, lung transplantation has been shown to confer a survival benefit for pwCF, but is limited by organ availability^11^.

Progress in CF has been aided by a strong global infrastructure supporting clinical care and accelerating research. **CF patient registries** of patient-consented demographic and clinical data are valuable resources for studying natural history/ long‐term health outcomes^12^, understanding impacts of treatments and informing future research. As examples, the UK CF Registry^13^ contains data from 10,655 people, an estimated 99% of individuals with CF in the UK under the care of specialist CF centres and clinics. The European Cystic Fibrosis Society Patient Registry (ECFSPR) (https://www.ecfs.eu/projects/ecfs-patient-registry/intro) was developed over a decade ago and now collects data from 38 countries and > 50,000 patients (www.ecfs.eu/projects/ecfs-patient-registry/annual-reports). **Clinical trial networks** have been key in catalysing clinical research for rare diseases. Several CF trials networks exist worldwide, the largest being the CF Foundation’s (CFF) Therapeutics Development Network (CFF TDN; https://www.cff.org/researchers/therapeutics-development-network) in North America. The European Cystic Fibrosis Society (ECFS) Clinical Trials Network (CTN; https://www.ecfs.eu/ctn) comprises 57 trials sites in 17 countries^14^. CTN works closely with national networks such as the UK CF Clinical Trial Accelerator Platform (https://www.cysticfibrosis.org.uk/the-work-we-do/clinical-trials-accelerator-platform) amongst others. **Patient organisations** (PO), such as the CFF (https://www.cff.org/), the UK CF Trust (https://www.cysticfibrosis.org.uk/) and the federation of European national CF Associations, CF Europe (https://www.cf-europe.eu/), are another key aspect of global CF infrastructure; several newer organisations are growing in regions such as the Middle East (https://www.mecfa.org/) and Latin America^15^. Regular engagement through these organisations builds strong, open relationships with the CF lay community enabling representation of and advocacy for people with CF and their families. The combined impact of the advances above led to mortality rates decreasing annually in the UK by 2% during 2006–2015. For pwCF born today survival into the fifth decade was predicted, even prior to the arrival of cystic fibrosis transmembrane conductance regulator (CFTR) modulators, the focus of the next section^16^.

#### The last few years: transformational change from CFTR modulator drugs

Building on this substantial progress in outcomes, the last decade has witnessed the development of new drugs targeting the basic defect in CF, with substantial health and quality of life benefits. The cause of CF is a reduction in the amount or function of CFTR protein at the surface of epithelial cells in the lungs and other organs. This directly reduces epithelial chloride and bicarbonate transport through CFTR and also perturbs function of a range of other ion channels and intracellular pathways with which CFTR interacts.

There are more than 350 recognised CF-causing mutations in the *CFTR* gene, from a total of >2000 identified variants (https://cftr2.org/). *CFTR* mutations have historically been grouped into six classes, based on how they impact CFTR transcription/ translation, intracellular trafficking or function. In practice, this distinction is not clear-cut, and the same mutation can cause defects in multiple aspects of CFTR expression^17^. For example, the most common mutation, found on at least one allele in around 90% of UK CF patients, is *p.Phe508del*, a deletion of three base pairs resulting in the deletion of phenylalanine at codon 508. This is generally regarded as a class II mutation leading to aberrant folding/ trafficking of CFTR and proteasome-mediated degradation; however, *p.Phe508del* CFTR also demonstrates reduced channel open probability (class III), and instability leading to rapid turnover at the cell membrane (class VI)^18,19^. Knowledge of different CFTR mutation classes has underpinned the development of new molecular therapies. An important initial step was the identification of ivacaftor, a CFTR potentiator that increases channel opening and ion transport^20^. This is highly effective in mutations where apical cell surface CFTR is present but dysfunctional (e.g., class III)^21^. Where mutations are mixed in class, such as *p.Phe508del*, ivacaftor has been combined with CFTR correctors, which improve folding, reduce protein degradation and facilitate protein trafficking to the cell surface. The initial dual drug combinations (lumacaftor/ ivacaftor and tezacaftor/ ivacaftor) led to modest clinical improvements in patients^22,23^, but most recently, a triple combination of two correctors targeting different aspects of CFTR misfolding (elexacaftor and tezacaftor) plus ivacaftor (ETI) has been developed. ETI is highly effective in patients with even just one copy of the *p.Phe508del* mutation, regardless of the second mutation^24–26^. This combination, licensed as Kaftrio® or Trikafta®, therefore offers an even greater possibility of effective CFTR modulation for ~ 90% of CF patients.

Clinically, CFTR correction can be measured by sweat chloride concentrations. In the ivacaftor and ETI trials, sweat chloride was reduced to levels below the diagnostic threshold for CF (60 mmol/L) in many subjects^25,26^. Trials of ETI have shown mean increases in ppFEV_1_ of around 14-15%^25,26^, beyond anything seen previously in CF. These drugs are also associated with other important clinical improvements, including in body mass index (BMI), reported well-being, and pulmonary exacerbation rates. There is also an increasing amount of data emerging from the use of CFTR modulators in routine clinical practice. The European Medicines Agency has strengthened the role of registries in data reporting by granting a favourable qualification opinion for the use of the ECFSPR as an appropriate data source for post-authorisation studies in monitoring effectiveness and safety of new drugs^27^; this will help ensure good quality evidence is gathered moving forward. Several large, real-world studies of ETI are underway. The PROMISE study, recently reported 6-month data, which largely mirrored efficacy and safety in clinical trials;^28^ improvements were observed in FEV1, BMI, quality of life and sweat chloride concentration. In Ireland and UK, the RECOVER study is underway, although at the time of writing, results have not been published. (clinicaltrials.gov NCT04602468)

However, drug costs are high: drug prices differ by geography and regional costs may not be fully transparent, but likely exceed £100,000 per patient annually in most regions. Drug discovery to proof of safety and efficacy in randomised controlled trials take up to 15 years and the rare disease space further drives high prices. Health technology appraisals by government bodies assess cost-effectiveness of new drugs, often using a cost per quality-adjusted life-year (QALY) in their deliberations. These economic evaluations require the use of health state utility values and the evidence for these in CF is sparse^29^. What monetary cost should be given to a longer or better-quality life is where controversy can arise, particularly when a new drug is not approved to be prescribed within a nation’s health care system.

#### Current status of drug development: clinical trials pipelines

**Fig. 2:**
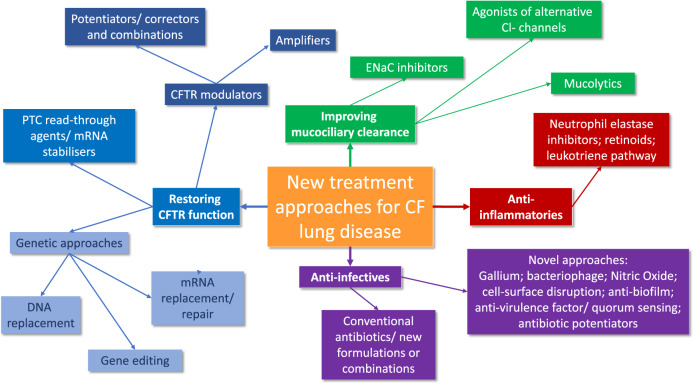
A large number of new approaches to CF therapy are progressing through from preclinical to clinical trial stages. Further detail can be found in the following review articles: CFTR modulator therapies^128^, genetic therapies^129^, mRNA-directed approaches and read-through agents^130^, mucoactive and airway hydrating drugs^131, 132^, anti-infectives^133^ and anti-inflammatories^134^.

Despite the therapeutic advances of the last few decades and the recent acceleration of these with modulator therapies, the community recognises that more will continue to be needed. It is encouraging that a pipeline of new drugs trials remains active and varied (https://apps.cff.org/trials/pipeline/; https://www.ecfs.eu/ctn/clinical-trials; https://www.cysticfibrosis.org.uk/get-involved/clinical-trials/trialstracker). In this section, we briefly outline progress to date in pulmonary therapies (Fig. 2 includes references for further reading) before focussing on remaining challenges and proposed research priorities.

There are many individuals who cannot be helped by current modulator therapy because their specific CFTR mutations are not amenable to the ‘making the most of a mutant protein’ approach, the hallmark of current modulator therapy. For these patients, alternative therapies are needed such as ribosomal readthrough agents, RNA-specific approaches (transfer (t)RNAs, mRNA stabilisers and repair). Both mRNA and DNA replacement are at clinical trial stages, whilst gene editing remains an area of active research.

For patients with class I (nonsense) mutations there is hope that small molecules will be identified that can facilitate premature truncation codon (PTC) read-through and/or impede mRNA decay allowing for clinically relevant levels of functional CFTR. Ribosomal read-through agents have a chequered history. Ataluren, the most extensively developed, was promising in preclinical studies and early trials but failed to demonstrate clinical benefit in larger phase three trials^30,31^. The drug is licensed for certain variants of Duchenne muscular dystrophy, but a recent systematic review confirmed modest impacts on disease progression, but flagged the need for further work on quality of life and cost utility^32^. Other ribosomal read-through drugs are in preclinical or early phase clinical trial stages^33,34^.

An alternative strategy to overcome PTC mutations is engineered transfer RNAs (tRNAs) (https://apps.cff.org/Trials/Pipeline/details/10162/ReCode-Therapeutics), designed to introduce an amino acid to an elongating peptide in place of the termination codon; early preclinical studies are encouraging^35^. PTCs lead to short-lived mRNA due to nonsense-mediated decay (NMD) mechanisms. General NMD inhibitors would be undesirable, as this is a normal gene expression regulatory mechanism, so specific antisense oligonucleotides are being developed; these stabilise mRNA leading to restored CFTR mediated chloride current in vitro^36^. Oligonucleotides to repair CFTR mRNA have also been explored. One such agent, Eluforsen, was a 33 base antisense oligonucleotide (ASO) targeting *p.Phe508del* CFTR. Treatment in vitro and in animal studies showed restoration of CFTR function using an array of electrophysiological assays^37^. In human trials, Eluforsen was well tolerated with a favourable safety profile and patients who were homozygous for the *p.Phe508del* mutation showed improvement in their nasal potential difference (NPD) a surrogate for CFTR restoration^38^. Whilst this drug is no longer being developed by ProQR, a similar approach is being used to target CFTR mutations that are more challenging to overcome such as splicing mutations and nonsense mutations. Splisense has several ASOs in preclinical development for 3849 + 10KbC > T and W1282X CFTR, which should reach clinical trials in the near future^39^.

All of the above will be specific to certain mutations which will disadvantage some, especially those with the rarest of variants. The development of ‘mutation agnostic’ treatments suitable for anyone with CF could be achieved with DNA or mRNA replacement strategies. the major challenge of which is delivery to the respiratory epithelium. An ongoing clinical trial investigating the potential of mRNA delivery for CF has reported interim results twice in the last eighteen months. Translate Bio uses a specialized lipid-based nanoparticle carrier for mRNA delivery (MRT5005) which is aerosolized for inhalation. The first interim results showed that subjects demonstrated a > 10% change in ppFEV_1_; however, this was not replicated in a subsequent treatment cohort. Other companies are taking a similar approach (https://apps.cff.org/Trials/Pipeline/details/10159/Arcturus-Therapeutics; https://www.cysticfibrosis.org.uk/news/vertex-announced-that-their-mrna-therapy-ind-has-been-cleared-by-the-fda). In terms of gene (DNA) transfer, to date, there have been several clinical trials of both viral and non-viral approaches, the vast majority of which were designed as early phase proof of principal studies without clinical efficacy read-outs^40^. The UK CF Gene Therapy Consortium (GTC) undertook a large, phase 2b trial of lipid-mediated *CFTR* gene transfer in 2014, reporting a statistically significant, but clinically modest, impact on FEV_1_^41^. Currently at the preclinical stage, the GTC is working in partnership with Boehringer Ingelheim and Oxford Biomedica towards a first-in-man trial using a pseudotyped lentiviral vector^42^. 4D Molecular Therapeutics and Spirovant are using adeno-associated vectors to transfer CFTR DNA and are currently at phase I and pre-clinical stages respectively (https://apps.cff.org/Trials/Pipeline/details/10161/4D-710; https://apps.cff.org/Trials/Pipeline/details/10160/Spirovant-Sciences). Despite decades of research, drugs that specifically target CF inflammation remain elusive. The inflammatory process in CF is complicated^43^, characterised by early and sustained neutrophil influx and high levels of elastase which correlate with structural damage on imaging even in early life. There is some evidence that normal resolution mechanisms are impaired^44^, so that inflammation can persist even if an infective insult is cleared^45^. There is certainly evidence that inflammation persists when CFTR function is corrected^46^. The presence of excessive neutrophilia, pro-inflammatory macrophages and a range of pro-inflammatory mediators provide numerous targets for therapeutic development^47^. One approach has been to target the neutrophil chemoattractant leukotriene B4 (LTB4) to inhibit neutrophil recruitment to the lung. Amebulant, an LTB4 antagonist, showed promise in preclinical evaluation but was associated with increased pulmonary exacerbations in a phase II study^48^. In contrast, the LTA4 hydroxylase inhibitor acebilustat (which reduces LTB4 levels rather than blocking the effect of LTB4 completely) was well tolerated and led to reduced lung neutrophil numbers in early phase studies^49^. It did not however meet its primary endpoint of improvement in FEV_1_, nor did it reduce exacerbations in a 48-week phase 2 trial^50^. The cannabinoid receptor agonist, lenabasum, reduces IL-6 transcription in macrophages in vitro^51^, suggesting direct effects on the inflammatory potential of these cells. A phase II clinical trial of lenabasum in pwCF showed modest clinical effects but a significant reduction in sputum interleukin-8^52^, and further studies are now underway. Despite significant effects on lung function, the impacts of CFTR modulators on lung inflammation are not yet known. A key early step in the process of tissue repair is the resolution of inflammation and the interaction of immune cells such as macrophages (known to be dysfunctional in CF) with resident stem cells may be key to this process^53,54^. Thus, the prospect of lung tissue repairing itself once CFTR function has been restored is intriguing. The ability of healthy lung tissue to regenerate after damage is well described and is contingent on the activity of basal cells in the airway (resident lung stem cells) that proliferate and differentiate in response to injury^55^. Whether this process occurs normally in CF is unknown but radiological improvements in inflammatory changes such as bronchiectasis have been reported in patients after prolonged modulator treatment^56^. We could speculate that by combining CFTR correction and anti-inflammatory strategies we may provide the perfect environment for tissue repair to thrive.

Infection (bacteria, mycobacteria and fungi) is a major problem in the CF lung. No published data are yet available on the impacts of ETI on infection, although there is a focussed substudy as part of PROMISE^28^. However, as many patients are already chronically infected at the time of commencing these therapies, we consider it likely that anti-infectives will continue to be required. The armamentarium available, limited both in scope and efficacy, means new agents must continue to be developed for these common organisms and also rarer, but more challenging pathogens such as the non-tuberculous mycobacterium (NTM) *M. abscessu*s^57^. A wide range of drugs is passing through the CFF TDN pipeline, including antibiotic adjuvants, biofilm targeted approaches and bacteriophages.

Airway surface rehydration and reducing mucus viscosity are additional targets of new therapeutic approaches. Sodium channel (ENaC) blockers have been under development for some time^58^, although none has yet progressed through pivotal trials to licensing^59^. An agonist of an alternative chloride channel, TMEM16A, termed EDT002 is in early phase trials (https://apps.cff.org/Trials/Pipeline/details/10172/ETD002)^60^. OligoG, a seaweed-derived oligonucleotide, acts both on mucus and bacterial biofilms and is currently in phase 2 trials^61^. Whether these agents will continue to be needed by patients experiencing substantial health benefits from CFTR modulators remains to be seen, but there certainly remains an unmet need in those for whom modulators are not appropriate; such drugs may also be useful outside CF for other forms of bronchiectasis and chronic obstructive pulmonary disease (COPD) for which treatments are currently lacking^62^.

### Current challenges in CF care

#### The growing CF population and changing care delivery needs

Improved health for pwCF has resulted in a significant increase in numbers of adults living with the condition^63^. With the introduction of CFTR modulators, morbidity and life expectancy are anticipated to improve further and the adult population will continue to expand in number for decades. Although respiratory and gastrointestinal morbidity may remain central to CF care (at least until children commence modulators very early in life), new and emerging complications associated with increased longevity means the CF care community must continually adapt and respond to the needs of this changing population. For example, microvascular complications including retinopathy, peripheral neuropathy and chronic kidney disease are now being identified in people with longstanding CF-related diabetes^64^, and patients who developed CF-related low bone mineral density in childhood/ early adult life are at greater risk of fracture from age-related bone loss, particularly following the menopause^65^. New health problems are also being identified as pwCF age, one example being an increased risk of gastrointestinal cancer, in particular of the bowel^66^. As a consequence, surveillance colonoscopy is now recommended for patients over the age of 40 years and in some regions for transplant recipients over the age of 30 years^67^. Although speculative, the high fat/high-calorie diet recommended for decades for pancreatic insufficient patients with CF may result in hypercholesterolaemia, type 2 diabetes and coronary artery disease in later life^68^. Even before the advent of CFTR modulators, pregnancy in female pwCF was increasing, and we expect this to continue^69^. The safety of modulators in pregnancy is now the subject of observational studies (Mayflowers, clinicaltrials.gov NCT04828382) and there will likely be a requirement for expanded antenatal and obstetric services for women with CF.

Following the introduction of CFTR modulators and the consequent reduction in exacerbation frequency and hospitalisation, the most appropriate model of care for pwCF is having to be reconsidered. The traditional outpatient model is time inefficient for healthcare teams and pwCF, and may not be well suited to those with mild or stable disease. Catalysed by the COVID-19 pandemic, virtual clinics using remote monitoring equipment (such as home spirometry, weight and oxygen saturation) and secure telephone/video conference platforms are becoming increasingly common^68^.

#### Remaining issues for those receiving CFTR modulator drugs

A number of issues remain, even for those people successfully established on modulators and demonstrating clinical benefits:

##### Disease will progress, albeit more slowly, and will be more challenging to monitor

Effective CFTR modulators will likely slow or at best, halt disease progression, but will not reverse a disease that has already become fixed, examples being pancreatic destruction in the majority, bronchiectasis and absence of the vas deferens. Pulmonary exacerbations still recur albeit less frequently, chronic infections are not fully cleared and there is persistent airway inflammation^46,70,71^. It is essential that we do not become complacent about disease progression in this population and yet monitoring will likely become more challenging. Effective surveillance for infection is critical in asymptomatic patients and underpins the management of young healthy children with CF who demonstrate disease progression despite a lack of symptoms^72–75^. Many patients previously able to expectorate sputum become non-productive on CFTR modulators, so pathogen surveillance will increasingly rely on alternative samples. Oropharyngeal sampling lacks sensitivity^76^ and bronchoalveolar lavage is invasive and time-consuming. Recently, interest has focussed on sputum-induction, which has been shown in several studies to outperform oropharyngeal sampling^77–79^. It is well tolerated, quick and relatively repeatable, even in young children. Similarly, spirometry may become less useful than more sensitive, but more time-consuming, tests such as lung clearance index or imaging.

##### Responses to treatments, including CFTR modulators, vary

There is considerable variability in patient response to modulators, much of which remains poorly understood, examples being FEV_1_ and sweat chloride responses. For lung disease, this may not be surprising: at least 50 candidate modifier genes have been proposed^80,81^, as well as several non-genetic influences on lung disease such as exposure to tobacco smoke and chronic *P. aeruginosa* infection^82,83^. Both of these may reduce modulator efficacy^84,85^. It seems likely that there will also be genetic influences on the absorption and metabolism of the drugs, an area ripe for further research^86^.

##### Adherence to CFTR modulators (and other treatments) may be challenging

Adherence to CF medications is often suboptimal with some studies suggesting that modulators may not be an exception in this regard^87,88^. For subjects experiencing great benefit, continuing to adhere to their symptom-directed therapies may be challenging; modulator trials to date have been ‘on top of’ such drugs and impact could be lessened if they are dropped. Advances are being made in supporting patients with adherence in other areas, such as mobile health applications, smart inhalers and chipped pill containers^89,90^.

##### Some people will experience side effects or tolerability issues

The long-term data available on ETI is limited, so we need to remain vigilant for emerging side effects, particularly those that are rare. Post-market surveillance and registry studies are invaluable in this regard. In addition to adverse effects reported in clinical trials, and in contrast to the group improvements in structured quality of life questionnaire scores, there is some evidence to link CFTR modulator therapies with worsening mental health in some individuals^91^, meaning adequate psychological and social support needs to be in place. Reporting of side effects in younger children is of paramount importance given the length of time they may be exposed to these agents.

#### Issues for patients not receiving CFTR modulators

Even in regions where modulators are funded, up to 10–15% pwCF do not have access to these drugs due to either unsuitable *CFTR* mutations or drug intolerances. Rather than confirmed as non-responsive, many *CFTR* mutations are simply so rare that they are poorly understood; ex vivo testing approaches may be helpful as discussed further in the next section. Other people may live in areas which have not yet approved reimbursement; the adverse impact of delayed access on health outcomes is substantial and has recently been modelled^92^. The team demonstrated that immediate access as compared with a 4-year delay would reduce the number of individuals with severe lung disease by 60%, increase the number with mild lung disease by 18% and reduce the number of pulmonary exacerbations by 19%. Over a 10-year period, deaths would be reduced by 15% and median age of survival increased by >9 years^92^. Over time therefore, the gap in physical health between people who are and are not receiving modulators will widen. In parallel, there may also be impacts on mental health and well-being if patients feel ‘left behind’. There are already trials of novel therapies targeted specifically at the non-modulator group and as these grow in number, competition for this small population may emerge and there may be perceived pressure to participate. In addition to those currently in the pipeline mentioned above, there is substantial preclinical work in genetic-based therapies such as gene editing^93–95^, which may in time translate into trials. This group of patients will also have an even greater need than the modulator-treated group for drugs targeting downstream consequences of CF such as anti-inflammatories, mucoactive agents or anti-infectives so it is essential we maintain momentum in these areas.

### Future directions & research priorities

#### Identifying priorities with the CF community

The James Lind Alliance Priority Setting Partnership (JLA PSP) in CF (https://www.jla.nihr.ac.uk/priority-setting-partnerships/cystic-fibrosis/), a list of top health priorities in CF, was compiled based on inputs from >600 contributors from >30 countries, including pwCF, family members and clinical care teams^96^. The 2017 partnership publication has been influential, stimulating both research and funding to address several of the priority questions. The top research priority in both the JLA PSP and in a large US survey was ‘reducing the burden of treatment’^97^. Focus on the role of the more burdensome conventional treatments (such as nebulised therapies) is increasingly relevant with the widespread availability of CFTR modulators. This is the foundation for the CF STORM (www.cfstorm.org.uk) and SIMPLIFY (clinicaltrials.gov NCT04378153) trials which aim to assess the real-world effects of stopping mucoactive agents; the latter has recently been published confirming non-inferiority of stopping either nebulised hypertonic saline or DNase over the short-term^98^. The CF community has also identified airway clearance techniques as the most burdensome of treatments;^99^ the question ‘Can exercise replace chest physiotherapy for people with CF?’ ranked highly^96^. Trial design here will be a major challenge, particularly in relation to ensuring compliance with the allocation arm and adherence to remaining treatments. The JLA PSP exercise was undertaken before the advent of increasing access to ETI. Priorities may now be very different for those with and without access to CFTR modulators. For this reason the CF JLA PSP has recently been refreshed (https://www.cysticfibrosis.org.uk/news/refreshed-top-10-research-priorities-for-cf-revealed).

#### Maintaining pace in development of- and access to- new therapies

The ECFS Task Force for speeding up access to new therapies has highlighted several priorities in this area^100–102^. Theratypes are groups of *CFTR* variants categorised according to their effect on CFTR protein^103^. Theranostics describes a personalised medicine approach to determine whether these variants respond to existing (or novel) drugs by pre-assessing agents directly on a patient’s tissue ex vivo^101^. This has been proposed as a pathway for testing of CFTR modulator therapies in individuals who are unlikely to be included in conventional clinical trials. There are multiple preclinical model systems for theratyping;^104^ in one example, spheroids are grown from epithelial cells (most commonly intestinal, but also respiratory) in which the apical cell surface faces into the lumen. Activation of CFTR leads to quantifiable swelling, which can serve as a personalised efficacy read-out for therapies restoring CFTR function^105^. The HIT-CF (www.hitcf.org) program is testing drug candidates on rectal organoids from pwCF with rare mutations. A cohort will then be selected for a clinical trial of various CFTR function restoring therapies. An ex vivo testing approach has already led the US Food & Drug Administration (FDA) to expand its licensed indication for modulators, although to date, the European Medicines Agency (EMA) has not adopted the same position.

Future clinical trials in CF will face very different challenges to those experienced to date^102^, likely different depending on whether they seek to recruit pwCF on or not on CFTR modulators. Those with mutations unsuitable for modulators represent generally small populations. Modifications to trial design may be required therefore to maximise power, such as adaptive designs, use of historical (including within-subject) controls, and seamless phase 2-3 protocols^106^. An in vitro theranostics approach as mentioned above may enable enrichment for those most likely to respond. Trial networks can prove valuable hub and spoke models allowing patients from these small subgroups to access trials being conducted outside their own centre^107^. Design of gene therapy studies poses some additional, specific challenges: there will be a need for long-term follow up, particularly for integrating viral vectors with a theoretical oncogenic risk (https://www.fda.gov/vaccines-blood-biologics/biologics-guidances/cellular-gene-therapy-guidances); previous participants could find themselves excluded from new trials of similar agents if immune response is a concern^108,109^. Providing comprehensive information as part of the consent process will be essential but is difficult in the face of known unknowns. Any currently plausible gene therapy/ editing approach will be topically targeted to the lungs, so there will not be the systemic benefits seen in some people on modulators. Finally, in terms of outcome measures, assays to confirm gene expression may be invasive (e.g., requiring bronchoscopy), prone to sampling error or lacking in sensitivity/ precision. As an example of the latter, even with ivacaftor, nasal potential difference measurements were a poor reflection of CFTR function in the sweat gland or of clinical benefit^110^, and the assay has not been used since in trials programmes for more recent modulators. With regard to clinical efficacy, the unprecedented success of ETI- in particular the large improvements achievable in FEV_1_ - has perhaps led to expectations for newer drugs which may be unrealistic and lead to disappointment. In our opinion, it would be unfortunate if a drug was rejected in its early stages based on more modest efficacy which still provided clinical benefit for this population with a high unmet need. When progression of a product is dependent on early/ mid-phase efficacy signal, academic investigators and commercial companies may differ in magnitude considered to be required.

In studies wishing to assess new drugs given to patients already receiving CFTR modulators a major challenge will be in assessing efficacy and selection of outcome measures. Patients receiving modulators are likely to have higher baseline FEV_1_, better BMI and fewer pulmonary exacerbations than previously^111^. This could mean that seeking further significant improvements in these clinical parameters is more challenging, requiring longer study times or larger patient numbers. Patient-reported outcome measures may also need to be revised, since they exhibit a ceiling effect in those with milder disease^112^. Alternative outcome measures may offer greater sensitivity to residual disease (Table 1). For example lung clearance index (LCI) has been especially useful to date in children and those with earlier-stage disease^113^. LCI has been used as a primary outcome in paediatric CFTR modulator studies, where FEV_1_ was within the normal range^114^, and will likely now find greater application in older patients on modulators. Chest computed tomography (CT) imaging provides detail about structural changes in the lungs and can provide proxy measures of small airway change such as gas trapping and mucus plugging^115^. The effect of widespread ETI treatment may also however make such findings harder to identify. New radiation-free magnetic resonance imaging (MRI) techniques, in particular those using hyperpolarised tracer gases to visualise ventilation distribution or other functional adjuncts such as oxygen-enhancement^116,117^, appear to be a sensitive measure of early airway change, and also provide regional detail. They may have a particular role in early phase studies or ‘n of 1’ trials in patients with unusual genotypes. Other outcome measures may need to be tailored to specific questions, such as gastrointestinal symptoms or imaging. If a study is placebo controlled, or requires washout of an existing modulator, recruitment may be an issue. Patients may not be willing to give up their proven modulators to trial an experimental drug with an uncertain chance of clinical efficacy^118^. Coming off modulators carries risks^119^, so placebo-controlled studies may be both ethically challenging and hard to recruit to. Short washout periods which are adequate for a change in sweat chloride and will minimise risk for patients may be useful in this context, with safety data collected subsequently from open-label, single arm extension phases^106^.

**Table 1:** Pros, cons and optimal use of current and future CF trial outcome measures

Outcome measure	Pros	Cons	Most suitable populations/trials
**Lung function**			
Forced expiratory volume in 1 second (FEV_1_)	• Performed routinely • Clinic/lung function lab or remote (home spirometer)	• Insensitive in mild lung disease • May need large numbers to detect small effects, particularly in people receiving modulators	• Adults or children with moderate to severe lung disease
Lung clearance index (LCI)	• Sensitive • Proven response to therapies • Standardised • Narrow normal range	• Time consuming • Operator dependent • Technology issues; different devices/ gases not interchangeable • Less useful in those with established disease	• Children, including pre-school • Adults with mild disease • Infants*
Impulse oscillometry	• Simple, non-invasive • Quick to complete • Alternative measures of small airways function	• Requires cooperation • Not well established in CF • Multiple measures generated • MCID unknown • Repeatability acceptable?	• Adults and older children
**Clinical endpoints**			
Exacerbation frequency	• Clinically relevant • Linked to survival and QoL	• Not fully standardised, subjective • Triggers variable and ill-defined • Much less common in pwCF on modulators	• Patients with higher frequency of exacerbations (e.g., >2/yr) • Phase 3 studies (long) • Real world evidence studies
CFQ-R respiratory domain score (patient-reported outcome measure)	• Well established • FDA-accepted outcome • Age and language-specific versions • Known MCID	• Ceiling effect with mild disease • Not tailored to post-modulator CF	• Those with more significant symptom burden • Adults • Non-modulator treated population
Weight/BMI & Height (Z score and absolute)	• Part of routine care	• Impact of non-CF factors	• Children (Z scores) and adults
Pancreatic exocrine function (FE-1)	• Can be assessed non-invasively	• Not recoverable if established pancreatic scarring	• Infants and children
Activity monitors	• Objectively measures physical activity in daily life • Technology easily accessible	• Not widely used • Likely to be highly variable and seasonal = require high numbers • Reproducibility/ responsiveness not established	• Phase 3 or real world evidence studies
Exercise capacity (e.g., VO2max)	• Associated with survival	• Equipment and time to perform • Ceiling effect in those with well-preserved lung function	• Older children and adults
Cough monitors	• Objectively measures cough in daily life • Standardised technologies	• Not widely used in CF • Patients may consider them intrusive • Reproducibility/ responsiveness not established	• Moderate/ severe disease • Early/ later phase trials • Real world evidence studies
**Chest imaging**			
High-resolution chest tomography (HRCT)	• Well established • Easily accessible • Fast • Standardised scores • Regional detail	• Radiation exposure • Scoring is operator dependent	• Children and adults • Trials involving long term outcomes
MRI, including ventilation-MRI	• Highly sensitive • Radiation free	• Limited availability • Long scan times • Low resolution for tissue morphology • Lack of standardisation	• Children and adults • Single or few-centre studies • Early phase trials • N of 1 trials
Mucociliary clearance	• Clinically relevant impact of CFTR correction	• Requires specialist equipment/expertise • Radiation exposure • Reproducibility/ responsiveness not fully established	• Adults and older children • Early phase trials as early indicator of efficacy
**Inflammatory markers**			
Sputum Inflammatory markers (e.g., neutrophil elastase, IL1, IL6 etc.)	• Objective • Associated with more severe disease	• Indirect biomarkers, no MCID • Sputum hard to sample in people on modulators • Noise: signal ratio likely high • Not consistently shown to change with treatment	• Early phase studies, proof of mechanism • Exploratory or secondary outcome measures
Blood Inflammatory markers (eg hsCRP, calprotectin)	• Objective • Associated with more severe disease	• Indirect biomarkers, no MCID • Little evidence of utility as trial outcome measure	• Early phase studies, proof of mechanism
**CFTR activity**			
Sweat test (chloride)	• Non-invasive • Direct measure of CFTR function; little impact of other factors • Standardised • Blinded	• Operator dependent	• All ages (technically challenging in small babies) • Phase I/II trials
Nasal potential difference	• Direct assessment of CFTR function within the airway • Standardised SOPs	• Technically challenging, requiring specialist equipment • Time consuming • Operator dependent • High degree of intra-subject variability • Poor correlation with other measures	• Older children and adults • Phase I/II trials
Intestinal organoids	• Direct assessment of CFTR function • Allows multiple drug combinations to be tested	• Invasive (rectal biopsy) • In vitro assessment • Highly specialised	• Phase I/II trials

*QoL* quality of life, *FDA US* Food and Drug Administration, *MCID* Minimal clinically important difference, *FE-1* faecal elastase-1, *IL* interleukin, *hsCRP* highly sensitive C-reactive protein.

*Available in highly specialised centres only.

Infants and children with CF make up the group with the chance of maximal benefit from new treatments for CF, as they may not already have irreversible organ disease. However, their advantage in this regard is also a problem in that measuring ‘improvement’ in any outcome measure from a ‘normal’ baseline is difficult. Children over the age of around 3 years are able to perform LCI and certain imaging modalities also show promise^113^. To date, however, regulatory approval for ivacaftor in the youngest cohorts has been based on pharmacokinetics, pharmacodynamics (sweat chloride) and safety, with efficacy extrapolated from older cohorts. Surprisingly large changes in faecal elastase, a biomarker of pancreatic exocrine function, have been demonstrated^120,121^, and this assay should be included in future trials of systemic CFTR-targeted approaches.

Given these challenges, it is essential that the patient community’s opinions are factored into trial design. The first step is to better understand what motivates people with CF to enrol into and remain in trials. A recent survey from Canada^122^, at a time when ~50% of the CF population had access to older modulator drugs but not yet to ETI, reported that adults with CF, or parents of children with CF, were most interested in trials that targeted the root or underlying cause of CF, inflammation and infection. In the UK, feedback from workshops facilitated by the CF Trust has revealed that pwCF are motivated to take part in research not only by the possibility of personal benefit but also in advancing treatment for others with CF and driving development of future options. Barriers to participation include adding to an already burdensome regime, fitting in around life (work, school, family), and avoidance of certain procedures. A UK based Delphi study confirmed these findings and identified a number of other aspects as facilitators or barriers^123^. Time required and missing out on other activities was a common theme, lending support for home-based or decentralised trial designs, for at least some visits. The COVID-19 pandemic has driven these developments faster than may have otherwise occurred^124^. Much is still to be learned regarding data/ sample quality, participants’ opinions and regulatory agencies’ positions, but we consider this type of approach an important consideration for some studies.

In addition to the patients’/ families’ involvement, collaboration between key stakeholders involved in the design, planning and delivery of clinical trials will improve efficiencies and increase chances of successful delivery. Interdisciplinary collaboration is particularly important for early phase translational studies where knowledge exchange is essential^125^. In the UK for example, the supporting research infrastructure includes our NIHR Respiratory Translational Research Collaboration (https://www.nihr.ac.uk/explore-nihr/support/respiratory-trc.htm), the National CF Research Strategy Group (authors of this article) and the CF Clinical Trials Accelerator Platform (https://www.cysticfibrosis.org.uk/the-work-we-do/clinical-trials-accelerator-platform); all of these are supported by the CF Community Involvement workstream of the CF Trust. Finally, the UK CF Registry has a well-established role in pharmacovigilance, including post marketing authorisation studies for safety and real-world effectiveness of CFTR modulators^70,126^. The registry can also support clinical trials with adaptations for eligibility assessment, recording consent, randomisation and outcome collection. It is currently hosting two randomised controlled drug trials, CF START (https://www.cfstart.org.uk/) and CF STORM (www.cfstorm.org.uk), both of which have been pragmatically designed to fit in with routine care, adding relatively little burden to either patient or clinical research team.

#### Addressing inequity in access to care

Authors of this article are largely from UK and deliver services in well-resourced healthcare systems. Even within the UK though, there have been prolonged periods of negotiation over new, high-cost drugs which in some cases have led to delays in access. These are nothing compared to the challenges faced by CF care teams in low and middle-income countries; even before high-cost CFTR modulators were developed, there were large differences globally in the provision of funding and expertise for CF care which was broadly reflected in survival^94,95^. For example, many patients in eastern European countries have limited access to specialist services and modulator therapies are not funded at all^96^. There is increasing recognition that CF is not the rarity it was previously considered in countries with non-white populations; many of these are in low and middle income brackets meaning the establishment of multidisciplinary services poses a major challenge. Rare *CFTR* mutations are commoner in these populations, posing a major unmet need with regard to modulator therapies. Even within high-income countries such as US and UK, patients’ and families’ socioeconomic factors are known to play a substantial role in disease expression of CF^95,97^, with particular impact in the early years of childhood;^98^ the impact- if any- of modulators on this gap requires research. Whilst tackling systemic, economic issues is a daunting challenge, there are ways in which the CF community can help: European Cystic Fibrosis Society (ECFS) has recently launched a twinning project (https://www.ecfs.eu/ecfs-standards-care/twinning), linking up highly experienced centres with those in less well-resourced areas for the sharing of clinical expertise, protocols and training materials. Participation in the ECFS Patient Registry where appropriate, may aid countries demonstrating unmet need to their funders. This was a major theme in the recent Lancet Respiratory Medicine Commission which took a global perspective on CF care, and in which several potential fruitful areas for future development are outlined^127^. As stated therein: ‘A concerted effort is needed to ensure that all patients with cystic fibrosis have access to high-quality health care in the future.’ Evidence-based standards of care guidelines produced by the European CF Society have facilitated access to resources across developing CF services.

### Conclusions

The collaborative working model adopted by the CF community to date, which has underpinned substantial progreess, needs to further continue to address health inequities and to ensure that the pipeline of new treatments remains active and successful. The NIHR Respiratory Translational Research Collaboration established the CF National Research Strategy Group specifically to identify areas of unmet need and provide potential solutions to challenges. The group of people with genetic variants unsuitable for CFTR modulator drugs remains a high priority and trials of genetic therapies will be a major focus of the next few years. The recent JLA Priority setting partnership (PSP) illustrated this, identifying treatment for this subgroup as the top priority research area. Knowledge arising from the PSP will be used to support the design and funding of both national and international research efforts to ensure that the progress made to date is further built upon, until (to quote the CF Trust) the hope for ‘a life unlimited’ by CF becomes a reality.
